# Surface Relief Gratings of Slide-Ring Hydrogels for Label-Free Biosensing

**DOI:** 10.3390/gels11060415

**Published:** 2025-05-30

**Authors:** Aitor Cubells-Gómez, María Isabel Lucío, María-José Bañuls, Ángel Maquieira

**Affiliations:** 1 Instituto Interuniversitario de Investigación de Reconocimiento Molecular y Desarrollo Tecnológico (IDM), Universitat Politècnica de València, Universitat de València, Camino de Vera s/n, 46022 Valencia, Spain; aicugo1@posgrado.upv.es (A.C.-G.); malube@upv.es (M.I.L.); amaquieira@qim.upv.es (Á.M.); 2 Departamento de Química, Universitat Politècnica de València, Camino de Vera s/n, 46022 Valencia, Spain

**Keywords:** hydrogel, label free, optical biosensing, slide rings, holography, surface relief grating

## Abstract

The creation of surface relief gratings using hydrogels for label-free biomolecule detection represents a significant advance in the development of versatile, cutting-edge biosensors. Central to this innovation is the formulation of materials with enhanced mechanical properties, especially for applications in soft, wearable technologies. In this work, we have developed novel biofunctional hydrogels that incorporate slide-ring supramolecular structures into their network, enabling the production of surface relief gratings with superior mechanical characteristics for biomolecule detection without the need for labels. These hydrogels, functionalized with bovine serum albumin and goat anti-rabbit antibodies, demonstrated excellent selectivity and sensitivity toward anti-bovine serum albumin and rabbit IgGs in blood serum, evaluated using a label-free format. Remarkably, the new materials matched the analytical performance of conventional hydrogels based on static networks while offering dramatically improved toughness and elasticity, with a compressive modulus comparable to human skin. This demonstrates the potential of slide-ring hydrogels for fabricating robust, label-free biosensing platforms. Furthermore, the flexibility of this system allows for the incorporation of various recognition elements tailored to specific applications.

## 1. Introduction

The demand for personalized medicine, aging populations, and the rise of chronic and infectious diseases are driving rapid growth in in vitro diagnostics (IVDs), particularly through point-of-care (POC) testing. In this sense, biosensors are presented as one of the approaches with more future projections in the field of IVD due to their high sensitivity, selectivity, and ability to work with minimal sample preparation [1]. But the spreading interest in biosensors is also related to their potential use in a wide variety of fields, such as the agri-food, industrial, and security sectors [2]. However, despite their potential, existing biosensors still face limitations in cost, portability, simplicity, and real-time performance, especially for on-site applications [3].

Label-free (LF) biosensors offer real-time, direct detection without altering the analyte, making them attractive for simple, cost-effective diagnostics [4,5]. Among LF biosensors, those devices based on holography, using diffraction gratings embedded in smart matrices, stand out due to their optical simplicity and responsiveness to refractive index changes [6].

Hydrogels, known for their biocompatibility and tunable properties, have proven especially suitable for such systems. Their porosity and ability to mimic physiological conditions make them ideal transducer materials for biosensing applications [7].

While various hydrogel-based holographic sensors have been reported, most target small molecules or ions [6]. Thus, the development of hydrogel-based holographic biosensors, understood as those that specifically detect an analyte through a particular bioreceptor such as nucleic acids, proteins, enzymes, aptamers, or even molecularly imprinted polymers, is still in its early stages [8,9,10,11]. Our group has contributed to this emerging field with hydrogel-based surface relief gratings (SRGs) for the detection of proteins and nucleic acids, achieving promising results even in complex samples like blood serum [12,13,14]. To address this, we present a new class of SRG biosensors based on **slide-ring (SR) hydrogels,** incorporating polyrotaxanes and exploiting their unique pulley-like molecular architecture for enhanced mechanical resilience [15,16,17]. Based on this principle, different materials have been manufactured that respond to stimuli such as temperature [18], pressure [19], or light [20], or with self-repairing properties [21], and they have even been engineered as wearable body-motion sensors [22,23].

In this study, we develop hydrogels incorporating polyrotaxanes, bovine serum albumin (BSA), and antibodies to fabricate mechanically robust surface relief gratings. These gratings are optimized for the direct, label-free detection of anti-BSA antibodies and rabbit IgGs. We conduct a comprehensive characterization of the materials, including their morphology, swelling behavior, and mechanical properties. In addition, the bioavailability of the anchored bioreceptors is assessed using fluorescence assays. Finally, we demonstrate the label-free biosensing capability of the system by monitoring changes in diffraction efficiency and the distances between diffraction orders.

## 2. Results and Discussion

### 2.1. Synthesis and Characterization of Hydrogels

**Scheme 1:**
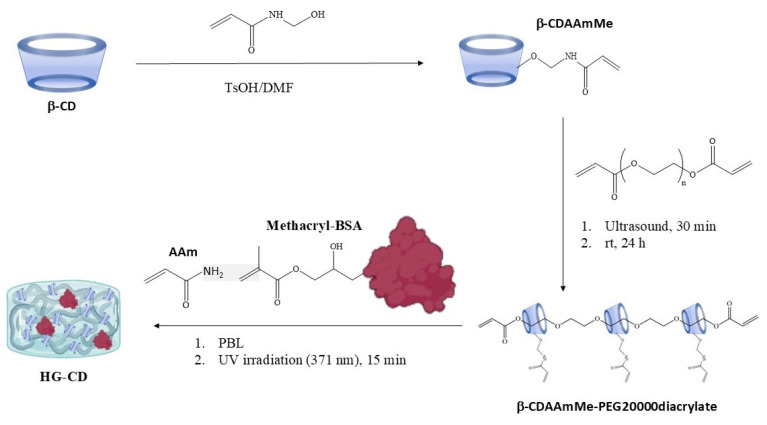
Reaction scheme to obtain slide-ring hydrogels (HG-CD): (1) synthesis of β-CDAAMe; (2) formation of polypseudorotaxane (β-CDAAmMe-PEG20000diacrylate); and (3) hydrogel polymerization (HG-CD).

**Figure 1:**
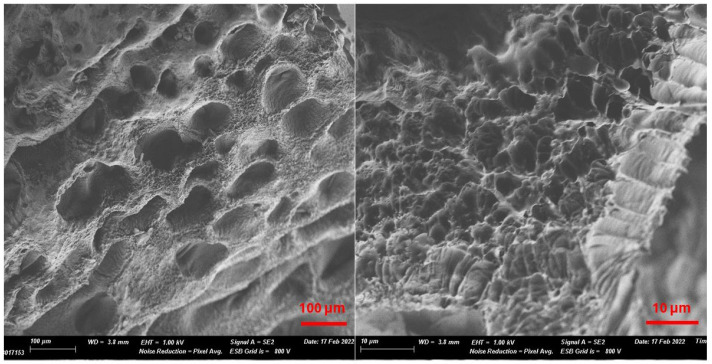
Scanning electron microscopy (SEM) images of hydrogel HG-CD-6 at different scales.

One of the main challenges faced when preparing surface relief gratings of bioresponsive hydrogel is their brittleness, which makes their handling difficult and hinders their extensive application. In order to improve the mechanical stability and elasticity of SRGs for biosensing applications, bioresponsive hydrogels based on the slide-ring principle were developed. For the synthesis of the hydrogels, acrylamide (AAm) was chosen as the main monomer in the polymerization to achieve stable and transparent materials. Additionally, sliding rings were included in the form of polypseudorotaxanes (β-CDAAmMe-PEG20000diacrylate) for the improvement of the hydrogels’ mechanical properties [15]. It is known that slide-ring polymers enhance the mechanical properties of hydrogels by allowing polymer chains to slide freely through movable crosslinks, called the pulley effect, leading to increased stretchability, toughness, and even self-healing capabilities while maintaining flexibility and resilience [15,16,17,21]. Also, the biorecognition element (BSA protein) was modified with methacrylate groups to afford its polymerization (methacryl-BSA) [13]. In this way, photopolymerization of the three components was conducted in water under 371 nm UV light irradiation using phenyl-2,4,6-lithium trimethyl-benzoylphosphinate (PBL) as a photoinitiator (Scheme 1). Our previous research advised us that the incorporation of proteins within the hydrogel matrix affects its consistency, which works against the correct further replica of the surface relief gratings and could even be detrimental to their transparency. Thus, different compositions of HG-CD with increasing concentrations of methacryl-BSA were tested (Table S1, Supplementary Materials). Among the various hydrogel formulations evaluated, HG-CD-6 was selected as the most suitable candidate for holographic optical biosensing based on a combination of critical performance criteria. It demonstrated excellent optical transparency, essential for accurate diffraction-based measurements, and exhibited consistent mechanical properties that allowed for successful fabrication of surface relief gratings via replica molding. Notably, both its transparency and structural integrity remained stable after immersion in PBS-T, indicating robustness under typical biosensing conditions (Figure S2, Supplementary Materials). Given these advantages, HG-CD-6 was chosen for further characterization, starting with an analysis of its micromorphology and porosity using scanning electron microscopy (Figure 1).

The material exhibits a morphology characterized by small pores of 2.51 ± 0.02 μm in size, which are organized into a structure forming medium-sized pores of 72.8 ± 0.8 μm (Figure S3, Supplementary Materials). In other words, the material features pores with an average size of 70 μm, while the walls of these larger pores contain a secondary porosity with smaller pores approximately 2 μm. The SEM analysis of similar hydrogels without polyrotaxane revealed a porous structure with a pore size of 26.03 ± 0.7 µm but with solid walls. In contrast, the newly developed hydrogels exhibit greater porosity, not only due to increased pore size but also due to the presence of porosity within the walls themselves. This high level of porosity gives the hydrogel a great capacity to absorb water and facilitates the entry and diffusion of the analyte within the matrix, which favors our biosensing purpose. The swelling kinetics of hydrogel HG-CD-6 in water were also characterized (Figure S4, Supplementary Materials). The maximum swelling degree reached by the hydrogel was 1223%. This swelling degree was higher than those obtained in our previous materials without slide-rings [13]. That would potentially endow them with better permeability and diffusion rates. Importantly, the introduction of polyrotaxanes improved the swelling capacity of hydrogels without ruining their plasticity.

**Figure 2:**
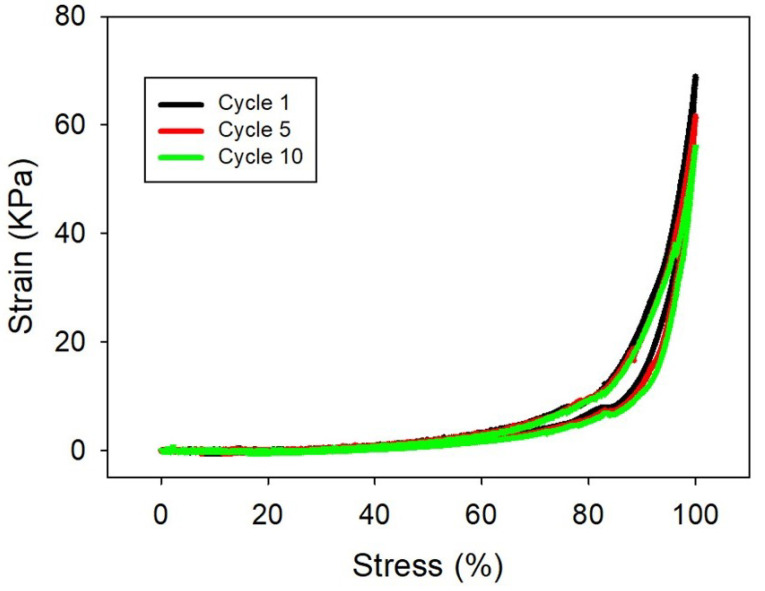
Stress–strain curves for HG-CD-6 hydrogel subjected to successive compression–release cycles.

Sliding rings are known to provide hydrogels with high elasticity, low hysteresis, and improved energy dissipation under deformation due to the described “pulley effect”, which allows the polymer to stretch more uniformly and efficiently compared to conventional polymers [16]. Therefore, HG-CD-6 hydrogel is expected to have a relatively low elastic modulus due to the presence of the polypseudorotaxanes. To corroborate this, a compression test was carried out with hydrogel HG-CD-6. Figure 2 shows the strain–stress curve when hydrogel HG-CD-6 was subjected to successive compression–release cycles. For clarity, only cycles 1, 5, and 10 are shown. Although the graphic shows certain hysteresis within the cycles, hydrogel HG-CD-6 can be totally compressed (almost 100% stress), and it totally recovered its initial state after the release of the compressive force (Video S1, Supplementary Materials). This process can be repeated, at least, up to 10 times without collapsing. Therefore, HG-CD-6 is a very tough and elastic material. Indeed, the compressive modulus calculated from the curve is 7.6 kPa. This value is in the same order of magnitude as human skin [24,25], so HG-CD-6 is a promising candidate for the design of wearable flexible sensors. Altogether, these results demonstrate that the introduction of slide-rings within the hydrogel matrix significantly enhances the mechanical properties of the SRG-aimed materials, thereby expanding their potential range of applications.

### 2.2. Ability to Specifically Recognize Anti-BSA by Fluorescence Assays

**Figure 3:**
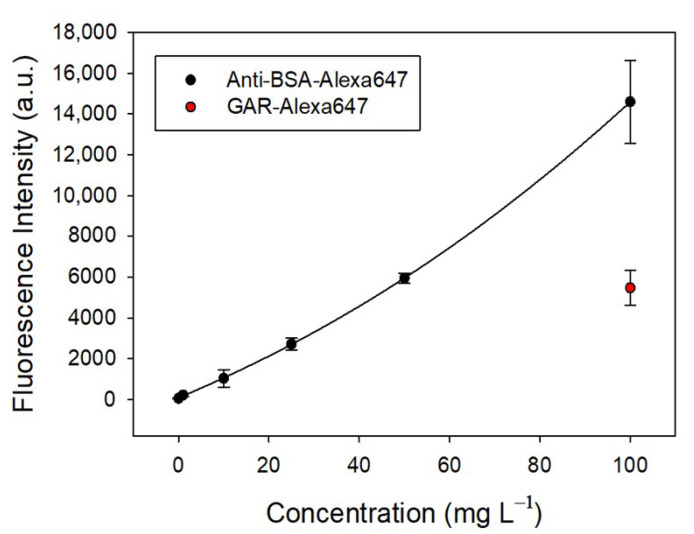
Fluorescence intensity of hydrogels incubated in increasing concentrations of anti-BSA-Alexa647 and GAR-Alexa647 for 30 min and washed in PBS-T for 30 min. Experiments were performed in triplicate, showing very good reproducibility.

Once the hydrogel was characterized, the presence of BSA anchored into the polymer in a bioavailable manner was assessed by fluorescence assays. HG-CD-6 was cut into pieces of 4 × 4 × 2 mm and incubated with increasing concentrations of labeled anti-BSA-Alexa647 or GAR-Alexa647 in PBS-T for 30 min. Fluorescence was registered after washing in PBS-T for 30 min. Figure 3 illustrates the fluorescence intensity of a hydrogel incubated with varying concentrations of Alexa647-labeled anti-BSA compared to a negative control (GAR-Alexa647). The fluorescence intensity of anti-BSA non-linearly increases with its concentration (exponential fit, R^2^ = 0.998), demonstrating a dose-dependent response. This trend suggests a strong and specific interaction between the hydrogel and anti-BSA, which becomes more prominent as the concentration rises. In contrast, the negative control (GAR-Alexa647) exhibits minimal fluorescence across all concentrations, with no significant change observed. This confirms the hydrogel’s specificity for anti-BSA and its lack of interaction with non-target proteins like GAR. The sharp increase in fluorescence intensity observed with anti-BSA at higher concentrations highlights the hydrogel’s ability to efficiently retain and bind the labeled antibody. The fluorescence intensity data, combined with the negative control, validate the hydrogel’s functional performance in recognizing and binding its target molecule, anti-BSA (Figure S5, Supplementary Materials).

### 2.3. Label-Free Biosensing of Anti-BSA-Sensing Hydrogel–CD

Once the ability of the hydrogel HG-CD-6 to selectively recognize anti-BSA was confirmed by fluorescence measurements, it was manufactured as a surface relief grating to use for label-free biosensing. The gratings were able to diffract light and to change the diffraction pattern under analyte recognition; thus, they were used as optical transducers.

**Figure 4:**
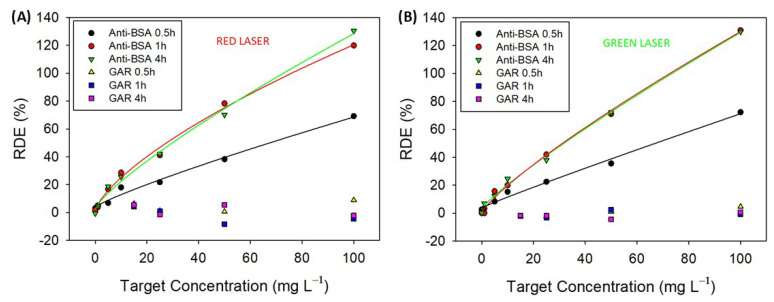
Binding curves for HG-CD-6 SRGs incubated with increasing concentrations of anti-BSA and GAR antibodies for different incubation times (0.5, 1, and 4 h) with (**A**) red (λ = 660 nm) and (**B**) green (λ = 532 nm) laser beams. All experiments were repeated three times for each concentration.

SRGs fabricated with HG-CD-6 were incubated with anti-BSA or GAR solutions at different concentrations, from 0 to 100 mg L^−1^, in PBS-T, and after incubation, the hydrogels were washed with PBS-T. Diffraction efficiency (DE (%)) of SRGs was registered before incubation and after incubation and washing using our homemade optical setup (Figure S1, Supplementary Materials). Various experimental conditions were investigated: (i) incubation for 4 h followed by overnight washing, (ii) incubation for 1 h followed by 1 h of washing, and (iii) incubation for 30 min followed by 30 min of washing. Additionally, two laser sources with wavelengths of 532 nm (green) and 660 nm (red), respectively, were utilized in the optical setup. Red light, with its longer wavelength, is less sensitive to small changes in the grating period compared to green light, which has a shorter wavelength and higher sensitivity to fine structural variations. Consequently, green light may provide a lower LOD and LOQ, enabling the detection of smaller changes in the hydrogel’s response. However, the choice of wavelength also depends on the hydrogel’s absorption and scattering properties, as well as the experimental setup, which can influence the signal-to-noise ratio and overall measurement precision. Figure 4 plots the relative diffraction efficiencies (RDEs (%)) calculated as indicated in Equation (3) for every experiment at different concentrations. Each point on the curve represents the measurement of three different SRGs, with diffraction efficiency recorded in three distinct areas for each SRG. The low error bars indicate the reproducibility of the manufacturing process. Additionally, since the SRGs are not produced in a single batch but are fabricated and stored over a two-week period, their stability is demonstrated, at least for this duration.

For all the studied conditions and laser sources, the RDE (%) values of the SRGs increased with the concentration of anti-BSA, while they remained practically invariable after incubating with GAR at the tested concentration range. In addition, there were almost no significant differences in the RDE (%) signals obtained with the red and green lasers for 4 h and 1 h of incubation. However, it was reduced by half when the incubation time was decreased to 30 min. A logistic correlation curve can best fit the data obtained for anti-BSA. Table S2 (Supplementary Materials) shows the limit of detection (LOD), the limit of quantification (LOQ), and IC_50_, together with the R^2^ values of the fitting, for every condition.

The limits of detection (LOD) and limits of quantification (LOQ) were determined based on the experimental calibration curves. The LOD and LOQ were calculated as the analyte concentration corresponding to the mean signal of ten blank measurements plus three or ten times their standard deviation, respectively. IC_50_ was defined as the anti-BSA concentration at which the relative differential extinction (RDE (%)) reached 50% of its maximum signal and was an indicator of the working interval for the biorecognition. The obtained LOD and LOQ values for both lasers improved with increasing incubation time and remained within the same order of magnitude as those previously reported for surface relief gratings (SRGs) fabricated using hydrogels without slide-ring crosslinks, but avoiding the associated brittleness of AAm/MBA hydrogels [13]. This demonstrates that incorporating slide-ring crosslinks significantly enhances the mechanical properties of the transducers without compromising their analytical performance. Remarkably, in HG-CD-6 hydrogels, the receptor concentration (BSA) was over 100 times lower than that used in hydrogels without slide-ring structures, yet comparable LOD and LOQ values were achieved. This is attributed to the presence of the movable crosslinks in the slide-ring polymer, which provide greater freedom of movement to the anchored bioreceptors, thereby enhancing their bioavailability. It represents a substantial improvement in terms of reagent efficiency, reducing both cost and resource requirements. IC_50_ values of HG-CD-6 SRGs were approximately 4-fold the values reached in hydrogels without sliding rings. Regardless of incubation time, a notable advantage was observed in the hydrogel’s saturation behavior and, thus, in the working interval. Unlike conventional hydrogels, the HG-CD-6 material did not reach signal saturation at any tested incubation time, enabling a broader dynamic range of detectable concentrations. This unique feature is likely attributable to the distinctive microstructure of slide-ring hydrogels, characterized by numerous cavities, as described earlier.

**Figure 5:**
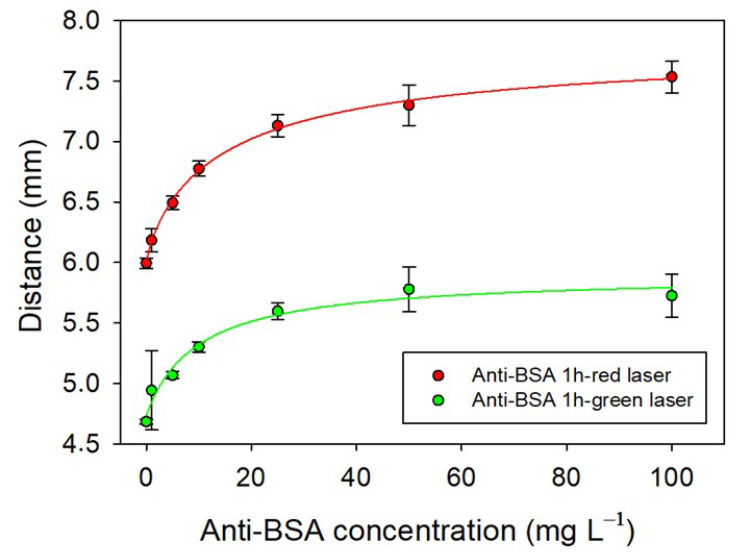
Distances between the diffraction spots of first and zero diffraction orders for the HG-CD-6 SRGs incubated with increasing concentrations of anti-BSA for 1 h and washed for 1 h with PBS-T. Data were obtained with red (λ = 660 nm) and b) green (λ = 532 nm) laser beams. All experiments were repeated three times for each concentration. Diffraction patterns were projected on a white screen placed 78 cm from the sample.

Additionally, distances between the orders of the diffraction patterns obtained from the illuminated surface relief gratings were used to monitor the response of the transducers to the target. When anti-BSA biorecognition occurs within the hydrogel matrix, it induces changes in the periodicity of the surface relief grating (SRG) lattice. These alterations affect the spacing between diffraction orders, which can be correlated with the analyte concentration. Polyclonal anti-BSA antibodies, capable of recognizing multiple epitopes on a single BSA molecule, may facilitate the binding of two antibodies to the same BSA. This interaction could lead to a compression of the grating structure, reducing its periodicity. Figure S6 (Supplementary Materials) shows the relation between the grating period and the distance between the diffraction spots of different orders of the orders that can be made using Bragg’s law. Thus, SRGs made with HG-CD-6 hydrogels were incubated with anti-BSA at increasing concentrations and washed with PBS-T, and the diffraction pattern obtained after their illumination with green and red laser beams and projected on a white screen was registered. Incubation and washing times of 1 h were selected as the best option from the diffraction efficiency experiments previously carried out [13]. Figure 5 shows that there is a direct relationship between the variation in the distance in the diffraction spots of the orders and the amount of anti-BSA, but a plateau is reached at concentrations higher than 25 mg L^−1^. Curves were fitted with a logistic fit, and LOD, LOQ, and IC_50_ were calculated as above (Table S3, Supplementary Materials). The LOD values are below 1 mg L^−1^, and a slight improvement in the LOQ is observed with the green laser. LOD and LOQ presented similar values to those obtained using RDE measurements for both lasers. The most significant difference was in the IC_50_ value, which in this case was lower, as the plateau was reached at 25 mg L^−1^. Probably, the limitation in this case is in the maximum distance that two antibodies can approach because binding the same BSA does not affect the mechanical capability of these types of materials. In any case, it was demonstrated that these materials also make it possible to monitor the concentration of anti-BSA simply by measuring the change in the distances between the diffraction orders. This measuring mode makes the reading and processing of data easier and simpler.

### 2.4. Detection of RIgG in a Rabbit Serum Sample

**Figure 6:**
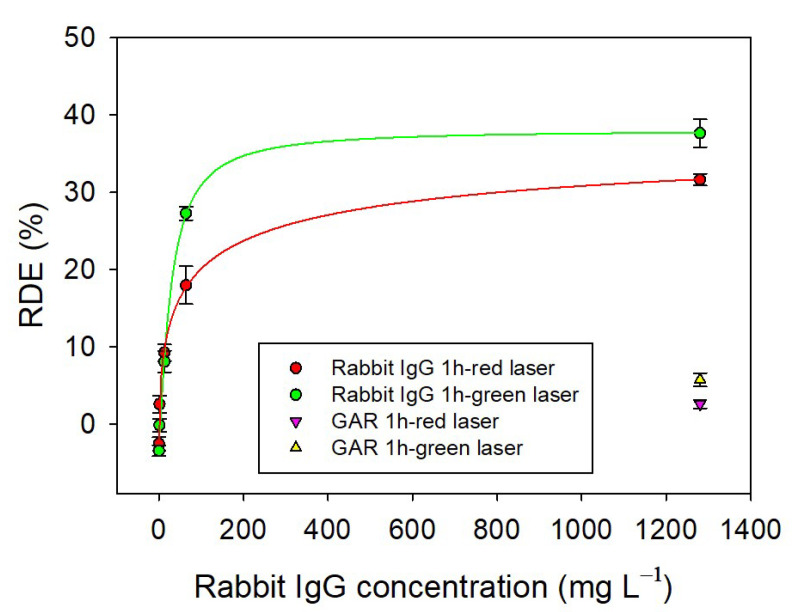
Dose-response curves for RIgGs-HG-CyD SRGs incubated with increasing concentrations of rabbit IgGs for 1 h after washing for 1 h with PBS-T. Measurements were carried out with green (λ = 532 nm) and red (λ = 660 nm) laser beams and were repeated three times.

The measurement of total immunoglobulin G (IgG) levels in serum is interesting in biosensing research, owing to its essential role in diagnostics, immunological surveillance, and therapeutic interventions. IgG is the most abundant antibody isotype in serum and serves as a critical biomarker for immune status, vaccine efficacy, and disease progression in conditions such as autoimmune disorders, infections, and cancers. Thus, broadening the applicability of our transducers to other biomarkers of interest, HG-CD-6 hydrogels were reformulated to include antibodies selective for rabbit immunoglobulins (RIgGs) that can be present in rabbit serum. SRGs were synthesized as before but included the composition of an optimized ratio of goat-anti-rabbit antibodies modified with methacrylate groups [21]. The selectivity of these SRGs to detect RIgGs was first studied by fluorescence assay. The synthesized RIgGs-HG-CD were incubated with increasing concentrations of rabbit IgG-Alexa647 (purified from serum by affinity column and labeled with Alexa647 according to the dye supplier’s instructions) and GAM-Alexa647 as the selectivity control for 1 h, and the fluorescence was measured after washing for 1 h with PBS-T. Figure S7 (Supplementary Materials) shows that the fluorescence intensity is proportional to the concentration of GAR-Alexa647, while the signal was much lower for GAM-Alexa647, which demonstrated a good selectivity towards the analyte of interest. RIgGs-HG-CD SRGs were used for the direct label-free detection of RIgG purified from rabbit serum. Figure 6 shows the RDE (%) of the RIgGs-HG-CD SRGs after 1 h of incubation with samples at increasing concentrations of rabbit IgG and 1 h of washing with PBS-T for both the red and green lasers. These conditions were chosen as they were the best in the assay for detecting anti-BSA. The RDE values of the SRGs increase with the concentration of purified RIgG, while they remain practically unchanged after incubation with GAR. Therefore, a specific response to purified RIgG in the concentration range tested was obtained for both lasers. The data can be best fitted using a logistic fit curve. The estimated values for the sensitivity, the limit of detection (LOD), and the limit of quantification (LOQ) were calculated as above and are shown in Table S4 (Supporting Information). The values for both lasers are aligned to those obtained for the detection of anti-BSA. The simplicity of the reformulation of the hydrogel composition envisages their utility to detect any type of IgG in serum just by changing the specific antibodies within its matrix. Although similar or alternative label-free approaches to the one presented in this work have been reported in the literature—many of them exhibiting highly competitive sensitivities—their implementation often involves complex fabrication processes and the use of sophisticated equipment. Moreover, a significant number of these methods do not perform detection in real matrices such as human serum. A comparative overview of the most recent approaches is provided in Table S5 (Supplementary Materials).

To validate the capability of our system in detecting IgG within real samples, we conducted a label-free biosensing experiment using raw rabbit serum. The serum was diluted at ratios of 1:10,000 and 1:200 with PBS-T. Concentrations were determined by interpolating the respective calibration curve (RDE (%) vs. RIgG concentration). The analysis revealed recovery rates of 75% and 70% for the 1:10,000 and 1:200 dilutions, respectively, which are slightly below the generally accepted immunoassay recovery range of 80–120%. These results can be attributed to the fact that the signals obtained are affected by the rest of the proteins and blood components. Thus, although detecting IgGs in real samples is possible with our system, additional improvements for quantitation at higher dilutions need to be made.

## 3. Conclusions

This study reports the development of mechanically robust hydrogel-based surface relief gratings incorporating slide-ring structures for the selective, sensitive, and label-free detection of anti-BSA antibodies and rabbit IgGs across a wide concentration range. The inclusion of slide-rings significantly improved the mechanical properties of the hydrogels without compromising their biosensing performance, enabling their potential use in flexible, wearable sensor applications. Fluorescence assays confirmed the specificity of the bioreceptors, while diffraction-based detection demonstrated clear, concentration-dependent responses with detection limits below 1 mg·L^−1^ and recovery rates of 70–75% in real serum samples. The platform also offers versatility for future adaptation to detect a broad range of analytes by immobilizing different recognition elements.

## 4. Materials and Methods

Bovine serum albumin > = 98% lyophilized powder (BSA), anti-bovine serum albumin antibody (anti-BSA), NHS Alexa Fluor^®^ 647 ester (succinimidyl ester), and goat anti-mouse IgG (H+L) cross-adsorbed secondary antibody labeled with Alexa Fluor™ 647 (GAM) were purchased from ThermoFisher Scientific (Madrid, Spain). Goat anti-rabbit antibody (GAR), acrylamide (AAm), glycerol, glycidyl methacrylate, dimethyl sulfoxide (DMSO), phenyl-2,4,6-lithium trimethyl-benzoylphosphinate (PBL), p-toluenesulfonic acid monohydrate, poly(ethylene glycol) diacrylate (PEG20000diacrylate), β-cyclodextrin (β-CD), and N, N-dimethylformamide (DMF) were purchased from Sigma-Aldrich (Madrid, Spain). Polydimethylsiloxane (PDMS) Sylgard 184 was obtained from Dow Corning (Wiesbaden, Germany). N-hydroxymethyl acrylamide was purchased from TCI (Madrid, Spain). PET-G masters with a period of 3 µm were fabricated by direct laser interference patterning (DLIP) as explained in Lucío et al. [12]. Buffers used: phosphate buffered saline (PBS1X, 0.008 M disodium phosphate, 0.002 M monosodium phosphate, 137 mM sodium chloride, and 2.7 mM potassium chloride, pH = 7.5); PBS-T (PBS1X containing 0.05% Tween 20); borate buffer (0.01 M borax, sodium hydroxide, pH = 9.5). All buffers were filtered through a 0.22 μm pore-size nitrocellulose membrane (Sigma-Aldrich, Madrid, Spain) prior to use. The ultraviolet (UV, λ = 371 nm) irradiation lamp was purchased from Jelight Company Inc. (Irvine, CA, USA). Anti-BSA and GAR antibodies were labeled with Alexa Fluor^®^ 647 following the supplier’s instructions to produce anti-BSA-Alexa647 and GAR-Alexa647. Methacryl-BSA and methacryl-GAR were synthesized as previously reported by us [13]. Rabbit immunoglobulins (RIgGs) were purified from rabbit serum (Sigma-Aldrich, Madrid, Spain) using HiTrap^®^ Protein G high-performance purification columns (Sigma-Aldrich, Madrid, Spain). Protein and antibody concentrations were determined with a NanoDrop 2000 spectrophotometer (Thermo Scientific, Madrid, Spain).

### 4.1. Synthesis of Slide-Ring Hydrogels (HG-CD-6)

Hydrogel–CD synthesis was carried out by adapting the protocol from Takashima et al. [26]. First, β-cyclodextrin (β-CD) was modified to incorporate polymerizable groups. A total of 15 g of β-CD and 2 g of hydroxymethyl acrylamide were dissolved in 100 mL of DMF. After adding 500 mg of p-toluenesulfonic acid monohydrate, the mixture was stirred for half an hour at 80 °C, and 500 mL of acetone was added. The resulting precipitate, β-CD modified with acrylate groups (β-CDAAmMe), was obtained after three washes and filtrates with 30 mL of acetone. Then, 200 mg of the obtained β-CDAAmMe was mixed with 50 mg of PEG20000diacrylate in 10 mL of distilled water, the resulting solution was sonicated for 30 min, and then stirred for 24 h at room temperature to generate a pseudopolyrotaxane (β-CDAAmMe-PEG20000diacrylate). Additionally, BSA protein was modified with acrylate groups following our reported protocol to yield methacryl-BSA [13]. Then, 1 mL of the pseudopolyrotaxane solution, 2.6 μL of methacryl-BSA in PBS1X (115.41 mg mL^−1^), and 4 mg of PBL were mixed with 3 mL of acrylamide in water (300 mg mL^−1^), and the solution was stirred for 15 min in the dark. Finally, 1.4 mL of the resulting solution was photopolymerized under UV light irradiation (λ = 371 nm) for 15 min in a dark chamber. All reaction steps are described in Scheme 1.

### 4.2. Fabrication of Antibody-Sensitive Hydrogel–CD SRGs

Anti-BSA-sensitive hydrogel–CD surface relief gratings (HG-CD-6 SRGs) were fabricated by replicating a PDMS mold obtained from a PET-G master with a 3 µm period. To produce the PDMS negative, a liquid PDMS mixture was poured onto the patterned side of the PET-G master, degassed under low vacuum for at least 15 min, and cured overnight at 60 °C. The cured PDMS mold was then peeled off and used as a template for SRG hydrogel fabrication. For this process, 1.4 mL of a homogenized mixture containing polypseudorotaxane (β-CDAAmMe-PEG20000diacrylate), methacryl-BSA, acrylamide, and PBL was drop-cast onto the structured surface of the PDMS mold affixed to a small vial (2 cm diameter). After a 10-minute vacuum purge, the mixture was exposed to UV light (371 nm) for 15 min to initiate polymerization. The formed hydrogel was peeled off and washed by immersion in PBS-T for at least 2 h, replacing the PBS-T three times to remove unpolymerized monomers. The resulting hydrogels were stored in PBS-T at 4 °C. Rabbit IgG-sensitive hydrogel–CD surface relief gratings (RIgGs-HG-CD SRGs) were synthesized following the same steps as for HG-CD-6 SRGs by adding 1.6 μL of methacryl-GAR (0.6 mg mL^−1^ in PBS1X). The details for the modification of methacryl-GAR have been previously described [13].

### 4.3. Mechanical Properties Characterization

Mechanical properties of the hydrogels were evaluated using a Mecmesin Multitest 2.5-i dynamic mechanical analyzer (Landes Poli Ibérica S.L., Barcelona, Spain). Hydrogels were cast in cylindrical molds measuring 85 mm in height and 90 mm in diameter and subsequently washed with PBS-T. Uniaxial compression tests were conducted at room temperature using a 25 N load cell, with a crosshead speed of 10 mm/min. The compressive modulus was determined from the stress–strain curves as the slope within the 35% to 45% strain range.

### 4.4. Scanning Electron Microscopy (SEM)

Hydrogel samples were characterized by SEM using a Gemini SEM 500 (Zeiss, Jena, Germany) system. First, hydrogels were swollen entirely in distilled water. Then, they were frozen at −20 °C. After that, they were dried for 12 h using a Telstar Lyoquest freezer (Terrassa, Spain). Finally, the dry aerogel samples were covered with a Pt layer (≈15 nm) using a BAL-TEC SCD 005 sputter coating (Leica Microsystems, Mannheim, Germany). The images were analyzed using the ImageJ 1.52a software to obtain the size distribution of the cavities of the hydrogels.

### 4.5. Swelling Studies

Hydrogels lyophilized with a size of about 1 cm^3^ were immersed in distilled water (20 mL) at room temperature to be swollen, and their weight was registered at different times. Before weighing, filter paper was used to remove the excess water from their walls. The swelling degree was calculated from Equation (1), where W_0_ is the weight of the lyophilized hydrogel and W_t_ is the weight of the hydrogel after its immersion in water for a time “t”.

(1) $$\%\;Swelling\;=\frac{W_{t}-W_{0}}{W_{0}}\times 100$$

### 4.6. Fluorescence Measurements

Prior to the fluorescence measurements, anti-BSA, purified rabbit IgG, and GAR antibodies were labeled with Alexa Fluor 647 dye following the protocol of the supplier to obtain anti-BSA-Alexa647, rabbit IgG-Alexa647, and GAR-Alexa647.

HG-CD-6 hydrogels were cut into 4 × 4 × 2 mm pieces and placed into a 96-well ELISA plate. They were incubated with 100 μL of anti-BSA-Alexa647 at concentrations of 1, 25, 50, and 100 mg L^−1^ in PBS-T for 30 min at room temperature. As a negative control, hydrogels were also incubated with GAR-Alexa647 at concentrations up to 100 mg L^−1^. After incubation, the solution was removed, and each well was washed with 300 μL of PBS-T for 30 min. Fluorescence was then measured using a custom-built surface fluorescence reader equipped with a CCD camera (λ_ex_ = 633 nm, λ_em_ = 670 nm) and quantified with Genepix Pro 6.0 software [27]. All experiments were performed in triplicate.

### 4.7. Diffraction Efficiency Measurements

A homemade setup equipped with a red laser (λ = 660 nm, 1.1 mW), green laser (λ = 532 nm, 1.6 mW), and two photodiodes was used to measure the diffraction efficiency (Figure S1, Supplementary Materials) [12]. All diffraction efficiency measurements were performed under specific conditions previously described [12]. An 8.2 KΩ photodiode was used to register the intensity of the zero-diffraction order (*I*_0_), and a 1.69 MΩ photodiode for the intensity of the first-diffraction order (*I*_1_). The diffraction efficiency of the gratings (*DE* (%)) was obtained from equation 2. The relative diffraction efficiency (*RDE* (%)) described in Equation (3) was used as the analytical signal to monitor the response of the hydrogels; *DE_i_* is the initial diffraction efficiency (before incubation with the analytes), and *DE_f_* is the final diffraction efficiency (after incubation and washing steps) for the first diffraction order. All experiments were repeated three times.

(2) $$DE\;\left(\%\right)\;=\frac{I_{1}}{I_{0}}\times 100$$

(3) $$RDE\;\left(\%\right)=\frac{DE_{f}-DE_{i}}{DE_{i}}\times 100$$

HG-CD-6 SRGs (dimensions: 4 × 4 × 2 mm) were positioned in a 96-well ELISA plate and covered with 100 µL of PBS-T. The initial diffraction efficiency (*DE_i_*) was measured prior to treatment. Subsequently, the hydrogels were incubated with 100 μL anti-BSA or GAR antibodies at increasing concentrations in PBS-T (0, 1, 10, 25, 50, and 100 mg L^−1^) for 4 h, 1 h, or 30 min. Following incubation, the hydrogels were washed with 300 μL of PBS-T overnight, 1 h, or 30 min, depending on the treatment. After washing, the supernatant was discarded, and 100 μL of fresh PBS-T was added for the final diffraction efficiency (*DE_f_*) measurements. The relative diffraction efficiency (*RDE* (%)) was calculated according to Equation (3). Each experimental condition was performed in triplicate.

The assays with the RIgGs-HG-CD SRGs were performed in a similar way. Samples of purified GAR (12.8 mg mL^−1^) were prepared at different dilutions in PBS-T (1:10,000, 1:1000, 1:200, and 1:10 dilutions). The *DE_i_*s of the RIgGs-HG-CD gratings were registered, and then they were incubated at room temperature with 100 μL of GAR dilutions for 1 h. Subsequently, the supernatant incubation solution was discarded, 300 μL of PBS-T was used for washing for 1 h, and, finally, the washing waters were removed before adding 100 μL of fresh PBS-T for the *DE_f_* measurements. *RDE* (%) was calculated according to Equation (3). As before, all experiments were carried out in triplicate.

Finally, RIgGs-HG-CD SRGs were used for the direct detection of IgGs in a real sample. For this, dilutions of 1:10,000 and 1:200 from unpurified rabbit serum in PBS-T were prepared. The DE measurements were conducted as before, keeping the same incubation times and washing steps.

## Data Availability

The original contributions presented in this study are included in the article/Supplementary Materials. Further inquiries can be directed to the corresponding author.

## Supplementary Materials

The following supporting information can be downloaded at: https://www.mdpi.com/article/10.3390/gels11060415/s1, Figure S1: Setup used to perform diffraction measurements; Table S1: Sample composition for 1 mL of hydrogel solutions; Figure S2: Photograph of hydrogel HG-CD-6; Figure S3: Histograms and distribution curves of cavity sizes; Figure S4: Swelling degree (%) of hydrogel HG-CD-6; Figure S5: Fluorescence image of hydrogels; Video S1: Mechanical compression of HG-CD-6 hydrogel; Table S2: Values of the limit of detection (LOD), the limit of quantification (LOQ), IC_50_, and R^2^ for HG-CD-6 SRGs obtained by label-free diffraction experiments; Figure S6: Diagram of the optical assembly; Table S3: Values of the limit of detection (LOD), the limit of quantification (LOQ), IC_50_, and R^2^ for HG-CD-6 SRGs obtained by measuring distances between the zero and first diffraction; Figure S7: Fluorescence intensity vs. rabbit IgG–Alexa647 concentration; Table S4: Values of the limit of detection (LOD), the limit of quantification (LOQ), IC50, and R2 for RIgGs-HG-CD SRGs; Table S5: Comparison of sensitivities and limits of detection for recently reported label-free optical biosensing approaches. References [8,9,10,28,29,30,31,32,33,34,35,36,37,38,39,40,41,42,43,44,45,46,47,48,49,50,51,52] are cited in the Supplementary Materials.

## Abbreviations

The following abbreviations are used in this manuscript:

AAm	Acrylamide
Alexa Fluor 647	NHS Alexa Fluor 647 ester (succinimidyl ester)
anti-BSA	Anti-bovine serum albumin antibody
BSA	Bovine serum albumin
CDs	Cyclodextrins
DE (%)	Diffraction efficiency
DLIP	Direct laser interference patterning
DMF	N,N-dimethylformamide
DMSO	Dimethyl sulfoxide
GAR	Goat anti-rabbit antibody
GAM	Goat anti-mouse IgG (H+L) cross-adsorbed secondary antibody
HG-CD	Hydrogel with cyclodextrin
HG-CD-6 SRGs	Anti-BSA-sensitive hydrogel–CD surface relief gratings
IgG	Total immunoglobulin G
IVDs	In vitro diagnostics
LF	Label-free
LOD	Limit of detection
LOQ	Limit of quantification
PBS1X	Phosphate buffered saline (
PBS-T	PBS1X containing 0.05% Tween 20
PBL	Phenyl-2,4,6-lithium trimethyl-benzoylphosphinate
PDMS	Polydimethylsiloxane
PEG20000diacrylate	Poly(ethylene glycol) diacrylate
POC	Point-of-care
PRs	Polyrotaxanes
RDE (%)	Relative diffraction efficiency
RIgGs	Rabbit IgG antibodies
RIgGs-HG-CD SRGs	Anti-rabbit IgG-sensitive hydrogel–CD surface relief gratings
SEM	Scanning electron microscopy
SR	Slide-ring
SRGs	Surface relief gratings
β-CD	β-Cyclodextrin
β-CDAAmMe	β-CD modified with acrylate groups
β-CDAAmMe-PEG20000diacrylate	Pseudopolyrotaxane
