# Supplementary material for: Surface Relief Gratings of Slide-Ring Hydrogels for Label-Free Biosensing
Source: Gels. 2025 May 30;11(6):415. doi: 10.3390/gels11060415 (PMC12191777; doi:10.3390/gels11060415)
Supplement: Supplementary file 1 [file gels-11-00415-s001.zip › gels-3647228-supplementary.pdf]

SUPPLEMENTARY MATERIALS

Article

Surface Relief Gratings of Slide-Ring Hydrogels for Label-free Biosensing

Aitor Cubells-Gómez [1], María Isabel Lucío [1,2], María-José Bañuls [1,2,*] and Ángel Maquieira [1,2]

[1] Instituto Interuniversitario de Investigación de Reconocimiento Molecular y Desarrollo Tecnológico (IDM), Universitat Politècnica de València, Universitat de València, Camino de Vera s/n, 46022 Valencia, Spain; aicugo1@posgrado.upv.es; malube@upv.es; amaquieira@qim.upv.es

[2] Departamento de Química, Universitat politècnica de València, Camino de Vera s/n, 46022 Valencia, Spain; malube@upv.es; amaquieira@qim.upv.es

* Correspondence: mbpolo@upv.es

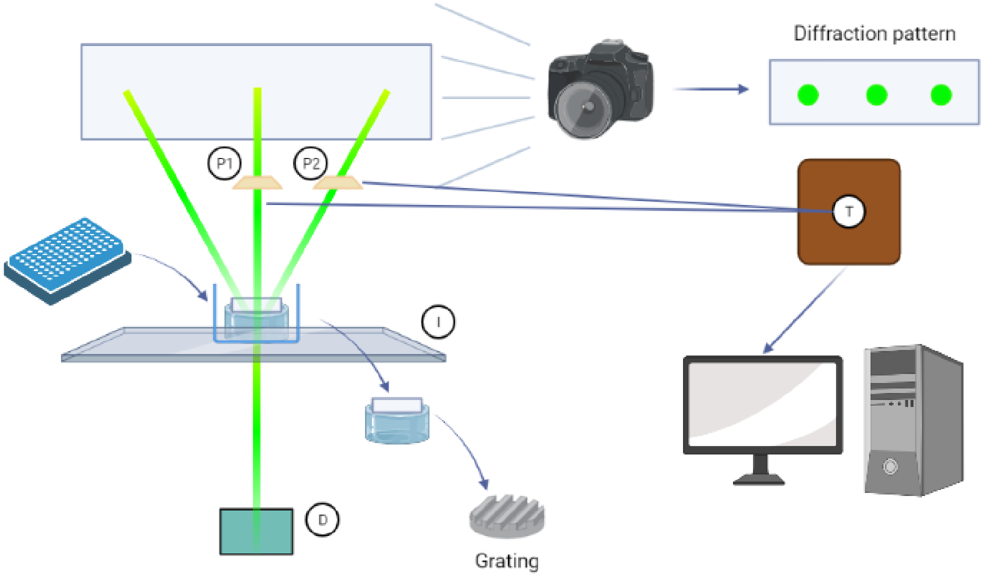

Figure S1. The set-up used to perform diffraction measurements. Where: D= laser provided with a diaphragm, l=moving lens, P1 and P2=photodiodes and T=transducer.

Table S1. Sample composition for 1mL of hydrogel solutions. [a] Methacryl-BSA: BSA protein modified with methacrylate groups at 115 mg mL^{-1} in PBS1X; [b] β-CDAAmMe-PEG2000 diacrylate: polypseudorotaxane at 5 mg mL^{-1} in water. [c] AAm: Acrylamide at 300 mg mL^{-1} in water and [d] PBL: phenyl-2,4,6-lithium trimethyl-benzoylphosfinate.

Samples	Methacryl-BSA (µL) [a]	β-CDAAmMe-PEG20000diacrylate (mL) [b]	AAm/H_2O (mL) [c]	PBL (mg) [d]	Transparency	Consistency	Buffer resistance
HG-CD-1	328.9	1	3	4	Low	Low	No
HG-CD-2	41.6	1	3	4	Medium	Low	Yes
HG-CD-3	20.8	1	3	4	Medium	Medium	Yes
HG-CD-4	10.4	1	3	4	High	Medium	Yes
HG-CD-5	5.2	1	3	4	High	High	Yes
HG-CD-6	2.6	1	3	4	High	High	Yes

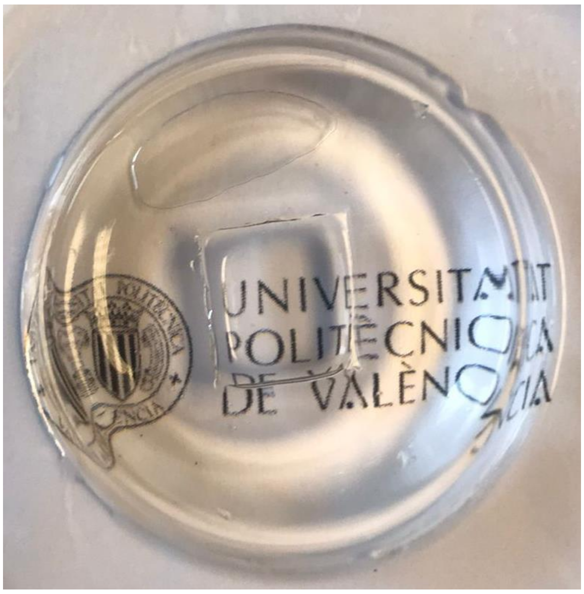

Figure S2. Photograph of hydrogel HG-CD-6, polymerized in a circular container provided with a square PDMS piece, after its immersion in PBS-T for 48 h. It shows a transparent and uniform appearance and potential malleability to further manufacture of surface relief gratings.

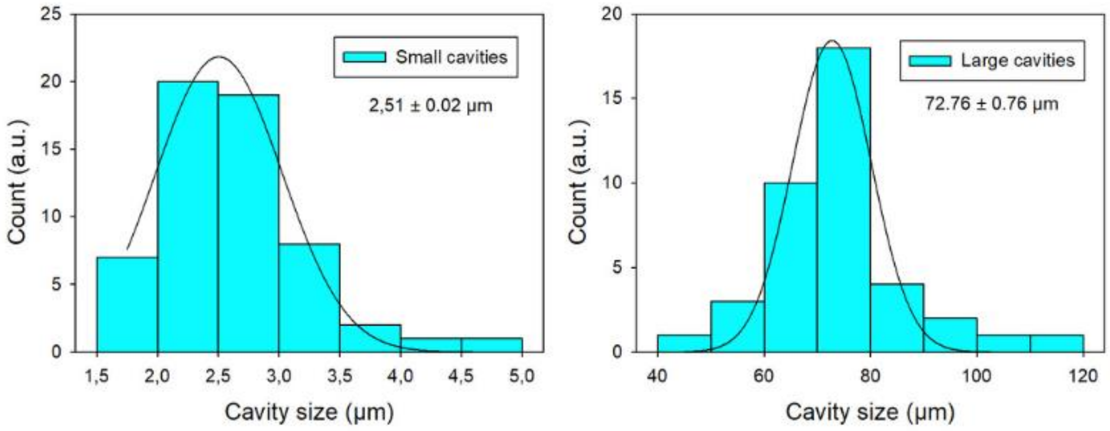

Figure S3. Histograms and distribution curves of cavity sizes for the different cavity populations were obtained from the scanning electron microscopy (SEM) images of hydrogel HG-CD-6.

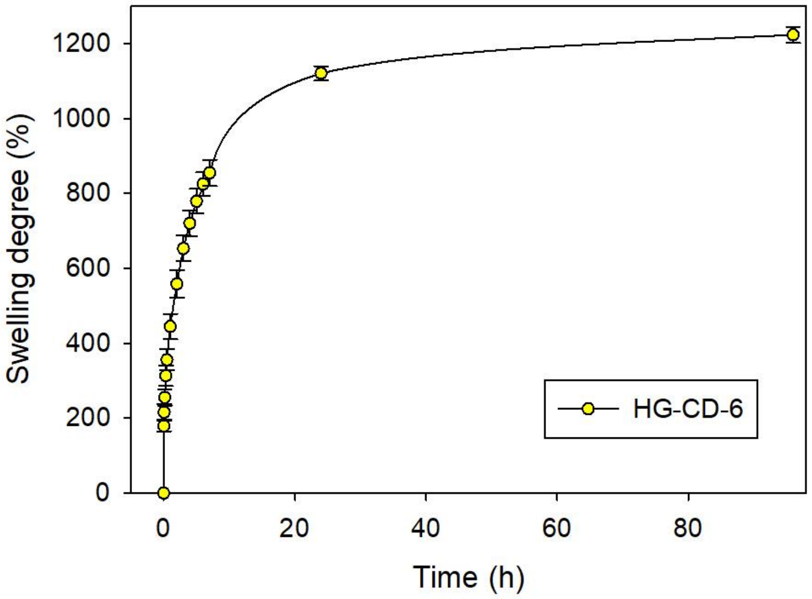

Figure S4. Swelling degree (%) versus water incubation time (swelling kinetics) of hydrogel HG-CD-6.

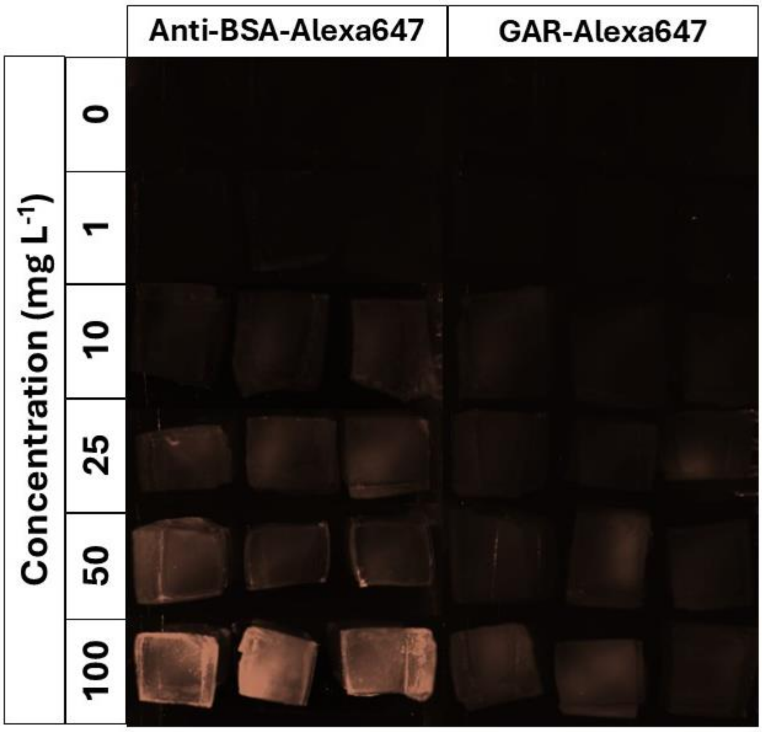

Figure S5. Fluorescence image of hydrogels incubated with increasing concentrations of labelled anti-BSA-Alexa647 and GAR-Alexa647 for 30 min after washing 30 min with PBS-T (λex = 633 nm, λem = 670 nm).

Table S2. Values of the limit of detection (LOD), the limit of quantification (LOQ), IC_{50}, and R^2 for HG-CD-6 SRGs obtained by label-free diffraction experiments carried out with green (λ= 532 nm) and red (λ= 660 nm) laser beams.

	RED LASER				GREEN LASER			
Incubation Time (h)	LOD (mg L^{-1})	LOQ (mg L^{-1})	IC_{50} (mg L^{-1})	R^2	LOD (mg L^{-1})	LOQ (mg L^{-1})	IC_{50} (mg L^{-1})	R^2
4	0.09	4.02	41.84	0.989	0.673	1.24	43.79	0.994
1	0.62	2.89	36.65	0.989	1.15	1.59	43.44	0.994
0.5	2.18	11.46	44.47	0.978	1.18	8.33	47.90	0.984

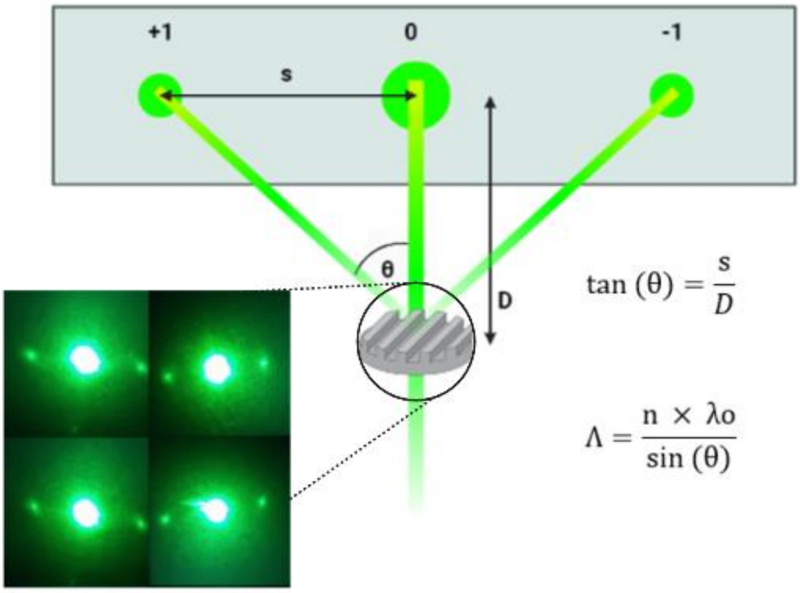

Figure S6. Diagram of the optical assembly used to demonstrate the relationship between grating periodicity and the distance between diffraction orders. Where: s=distance between a given order and order 0, D=distance from which the images were obtained, n=order, λ_0=incident laser wavelength (λ_{green}= 532 nm or λ_{red}= 660 nm), Λ=network period and θ=angle resulting from the laser beams corresponding to a given order 1 and order 0.

Table S3. Values of the limit of detection (LOD), the limit of quantification (LOQ), IC_{50}, and R^2 for HG-CD-6 SRGs were obtained by measuring distances between the zero and first diffraction of their diffraction patterns.

	RED LASER				GREEN LASER			
Time (h)	LOD (mg L^{-1})	LOQ (mg L^{-1})	IC_{50} (mg L^{-1})	R^2	LOD (mg L^{-1})	LOQ (mg L^{-1})	IC_{50} (mg L^{-1})	R^2
1	0.82	4.04	13.84	0.997	0.86	2.60	8.96	0.997

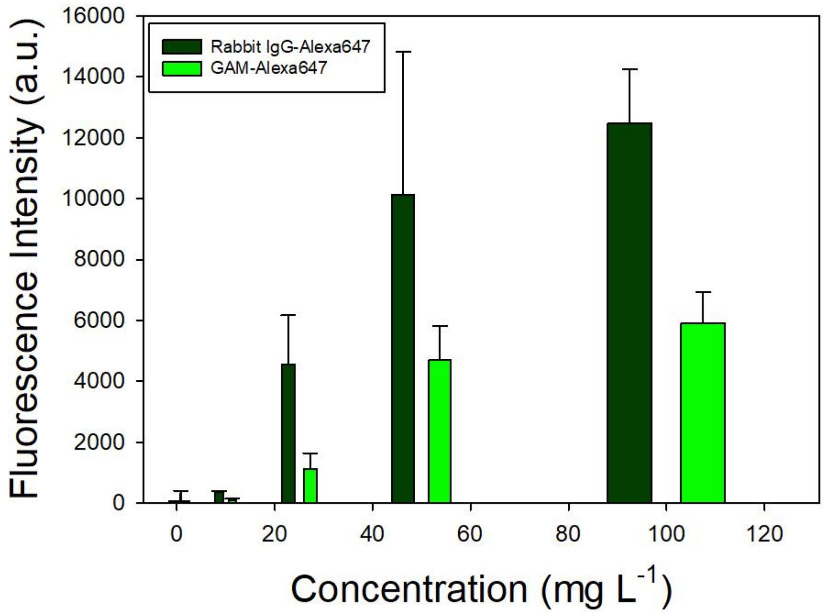

Figure S7. Fluorescence intensity increases proportionally with the concentration of rabbit IgG–Alexa647, while remaining low for goat anti-mouse (GAM)–Alexa647, demonstrating high selectivity for the target analyte. Notably, the initial fluorescence of the GAM–Alexa647 solution at 100 mg/L was approximately 40% higher than that of rabbit IgG–Alexa647 (49,301 a.u. vs. 30,177 a.u.), explaining the elevated background signal observed in the negative control.

Table S4. Values of the limit of detection (LOD), the limit of quantification (LOQ), IC_{50}, and R^2 for RIgGs-HG-CD SRGs obtained with green (λ=532 nm) and red (λ=660 nm) laser beams.

	RED				GREEN			
Time (h)	LOD (mg L^{-1})	LOQ (mg L^{-1})	IC_{50} (mg L^{-1})	R^2	LOD (mg L^{-1})	LOQ (mg L^{-1})	IC_{50} (mg L^{-1})	R^2
1	0.3	5.47	63.02	0.999	1.4	6.19	34.99	0.983

Table S5. Comparison of sensitivities and limits of detection for recently reported label-free optical biosensing approaches similar or alternative to the present work.

Analyte	Sample	Sensitivity/LOD	Measurement Approach	Ref
Bovine immunoglobulin (BIgG)	buffer	0.5 μg mL^{-1}	Nanoporous Polystyrene Inverse Opal Materials with Optical Interference	1
Chagas specific antibodies present in the blood of patients	clinical serum samples	60 μg mL^{-1}	Nanograting gold plasmonic sensor	2
Yersinia pestis (agY) antigen for plague diagnosis	buffer	~14.2 pg mL^{-1}	Dual-stripe nanowire arrays	3
Cardiac myoglobin	buffer	49 ng mL^{-1}	Silicon nitride gratings	4
SARS-CoV-2 antigen	buffer	62 pg mL^{-1}	Multilayer Fabry-Perot-type interference	5
Hemoglobin	DIW	0.057 mg mL^{-1}	Micro-tapered long-period fiber grating	6
Pseudomonas exotoxin A	buffer	0.21 ng mL^{-1}	Optical fiber biosensor with a bilayer bio-sensitive film	7
Lactose	milk	0.005 wt%	Optical bi-enzyme-titania reactive strip	8
Urea	buffer	0.36 mM	Amorphous photonic films with urease modified hydrogel	9
DNA fragments from the human citomegalovirus/ antibodies against SARS-CoV-2	buffer/ human plasma	4.2 pg mm^{-2}	Imaging Diffractometric	9
Glucose	urine	0.0020 dL mg^{-1}	Photonic waveguide	10
Antibodies specific to cancer biomarker CSPG4.	buffer	nr	Surface Plasmon Resonance (SPR)	11
BSA/antiBSA	buffer	6 pg mm^{-2}	Strong plasmonic gratings	12
Folic acid (indirect competitive)	buffer	0.22 nM	Topologically dark metamaterials with scissor effect	13
SARS-CoV-2 spike protein	buffer/saliva	3.05 ng mL^{-1}	Combined fiber multi-mode interference with grating resonance	14
Pseudomonas aeruginosa bacteria	buffer	89 CFU mL^{-1}	Guided mode resonance	15
Streptomycin	buffer	0.40 nM	Liquid crystal-infiltrated photonic crystal sensing film	16
Streptavidin	buffer	nr	Optofluidic Young Interferometer	17
Kanamycin	milk	1.10 pg mL^{-1}	2D photonic crystal	18

Anti-SARS CoV-2 antibodies	human serum	n r (discrimination between positive and negative)	1D photonic crystal	[19]
Cocaine	buffer	$2.15 \cdot 10^3$ μg mL^{-1}	Bragg Diffraction Grating Wavelength Shift	[20]
Human Thrombin	buffer	2.42 mg^{-1} mL	Surface Relief Grating Diffraction Efficiency	[21]
Human IgG	buffer	3.21 μg mL^{-1}	Surface Relief Grating Diffraction Efficiency	[22]
Human IgG	serum samples	1.95 μg mL^{-1}	Surface Relief Grating Diffraction Efficiency	[23]
Anti-BSA	serum samples	0.1 μg mL^{-1}	Optical Fiber Bio Bragg Gratings Reflectivity	[24]
Anti-BSA	buffer	0.3 μg mL^{-1}	Rib Waveguide Bio Bragg Gratings Reflectivity	[25]
C-reactive protein	serum samples	1.07 μg mL^{-1}	Surface Relief Grating Diffraction Efficiency	[26]
Testosterone	buffer	0.29 μg mL^{-1}	Surface Relief Grating Diffraction Efficiency	[27]
Anti-SARS-CoV-2	serum samples	19.9 ng mL^{-1}	Surface Plasmon Resonance (SPR)	[28]
Anti-SARS-CoV-2	serum samples	0.1 μg mL^{-1}	Nanoplasmonic Refractivity Imaging	[29]
HER2 biomarker	buffer	0.66 μg mL^{-1}	Optical Fiber-based SPR	[30]
Anti-BSA	buffer	0.4 μg mL^{-1}	Surface Relief Grating Diffraction Efficiency	[31]
Anti-BSA	buffer	0.17 μg mL^{-1}	Surface Relief Grating Diffraction Order Distance	[31]
AntiBSA/ Rabbit IgG	buffer/ serum	0.09 μg mL^{-1}/ 0.3μg mL^{-1}	Surface Relief Grating Diffraction Efficiency	this work
AntiBSA	buffer	0.82 μg mL^{-1}	Surface Relief Grating Diffraction Order Distance	this work

References:

(1) Wang, T.; Wang, L.; Ma, N.; Zhang, Y.; Liu, L.; Wan, Y.; Zhou, L.; Qian, W. Nanoporous Polystyrene Inverse Opal Materials with Optical Interference Properties for Label-Free Biosensing. *Langmuir* **2024**. https://doi.org/10.1021/ACS.LANGMUIR.4C01947/SUPPL_FILE/LA4C01947_SI_001.PDF.

(2) Chain, C. Y.; Prieto, E. D.; Cisneros, J. S.; Daza Millone, M. A.; Ramirez, E. A.; Labriola, C. A.; Aniasi, V.; Villagra, A. P. M.; Franchin, L.; Paccagnella, A.; Bonaldo, S. Plasmonic Biosensor of Cruzipain-Antibody Complexes Based on Gold Nano-Gratings for Chagas Disease Screening. *IEEE Sens J* **2025**. https://doi.org/10.1109/JSEN.2025.3528721.

(3) Chen, J. K.; Zeng, X. Y.; Chang, C. J.; Chen, C. W. Poly[2-(Dimethylamino)Ethyl Methacrylate]/Gold Nanoparticle Composite Dual-Stripe Nanowire Arrays as Optical Biosensors for Label-Free Plague Diagnosis. *J Taiwan Inst Chem Eng* **2023**, *146*, 104855. https://doi.org/10.1016/J.JTICE.2023.104855.

(4) Beliaev, L. Y.; Takayama, O.; Xiao, S. Effectively Detecting Cardiac Myoglobin by Use of Bound States in the Continuum in Silicon Nitride Gratings. *J Appl Phys* **2024**, *135* (22), 223101. https://doi.org/10.1063/5.0208969/3297609.

(5) Wang, Y.; Li, Z.; Li, X.; Gao, K.; Yin, Z.; Liu, W.; Zhong, B.; Kan, G.; Wang, X.; Jiang, J.; Shen, Z. Highly Absorbing Monolayer MoS2 for a Large Reflection Phase Modulation. *Adv Opt Mater* **2024**, *12* (25), 2400429. https://doi.org/10.1002/ADOM.202400429.

(6) Li, Y.; Du, M.; He, S.; Wang, R.; Zhang, Z.; Wang, Q. Sensitive Label-Free Hemoglobin Detection Based on Polydopamine Functionalized Graphene Oxide Coated Micro-Tapered Long-Period Fiber Grating. *Optik (Stuttg)* **2023**, *275*, 170626. https://doi.org/10.1016/J.IJLEO.2023.170626.

(7) Guo, W.; Yu, Y.; Jin, G.; Xin, C. Optical Fiber Biosensor with a Bilayer Bio-Sensitive Film for Label-Free Detection of *Pseudomonas* Exotoxin A. *Chinese Optics Letters, Vol. 22, Issue 11, pp. 111201-* **2024**, *22* (11), 111201-.

(8) Shavronskaya, D. O.; Nazarova, E. A.; Krivoshapkina, E. F. Optical Bi-Enzyme-Titania Biosensor System: A New Way to Detect Lactose. *Biosens Bioelectron X* **2023**, *14*, 100347. https://doi.org/10.1016/J.BIOSX.2023.100347.

(9) Han, P.; Li, Y.; Zhao, B.; Li, H.; Wang, Z.; Liu, X.; Meng, W. Visual and Label-Free Detection of Urea Based on Amorphous Photonic Films with Non-Iridescent Structural Colors. *Anal Chim Acta* **2025**, *1345*, 343731. https://doi.org/10.1016/J.ACA.2025.343731.

(10) Sinha, S.; Panda, T. K.; Sarkar, P.; Palai, G.; Pal, M.; Kumar, B. A.; Mohanty, S. K. Designing Efficient Photonic Waveguides for Glucose Detection in Human Urine for Diabetic Management. *Journal of Optics (India)* **2025**, 1–13. https://doi.org/10.1007/S12596-025-02683-X/FIGURES/9.

(11) Hasler, R.; Cattozzo Mor, D.; Aktug, G.; Fossati, S.; Vu, V. T.; Tamayo, A.; Giordani, E.; Ricciardi, E.; Giacomini, P.; Perutka, J.; Onder, K.; Kleber, C.; Samorì, P.; Huang, C. J.; Dostalek, J. Surface Plasmon Resonance Biosensor with Anti-Crossing Modulation Readout. *Sens Actuators B Chem* **2024**, *417*, 136163. https://doi.org/10.1016/J.SNB.2024.136163.

(12) Laffont, E.; Valour, A.; Crespo-Monteiro, N.; Berini, P.; Jourlin, Y. Biosensing in the Optical Switch Configuration on Strong Plasmonic Gratings Enabling Differential Referenced Detection. *Sens Biosensing Res* **2024**, *45*, 100681. https://doi.org/10.1016/J.SBSR.2024.100681.

(13) Tselikov, G. I.; Danilov, A.; Shipunova, V. O.; Deyev, S. M.; Kabashin, A. V.; Grigorenko, A. N. Topological Darkness: How to Design a Metamaterial for Optical Biosensing with Ultrahigh Sensitivity. *ACS Nano* **2023**, *17* (19), 19338–19348. https://doi.org/10.1021/ACSNANO.3C06655/ASSET/IMAGES/LARGE/NN3C06655_0004.JPEG.

(14) Bae, M.; Choi, S.; Kim, J.; Seo, G.; Lee, Y. W. Temperature-Insensitive Label-Free SARS-CoV-2 Spike Protein Detection Based on Complementary Refractive Index and Temperature Dependence of Multi-Mode Interference and Grating Resonance. *Talanta* **2024**, *266*, 125091. https://doi.org/10.1016/J.TALANTA.2023.125091.

(15) Joseph, S.; Rajpal, S.; Kar, D.; Devinder, S.; Pandey, S.; Mishra, P.; Joseph, J. Guided Mode Resonance Immunosensor for Label-Free Detection of Pathogenic Bacteria Pseudomonas Aeruginosa. *Biosens Bioelectron* **2023**, *241*, 115695. https://doi.org/10.1016/J.BIOS.2023.115695.

(16) Zhu, A.; Gong, W.; Bu, D.; Zhou, J.; Wu, Z.; Yu, R. Stable Liquid Crystal-Infiltrated Photonic Crystal Sensing Film for Facile Detection of Streptomycin. *Biosens Bioelectron* **2025**, *275*, 117225. https://doi.org/10.1016/J.BIOS.2025.117225.

(17) Labella, E.; Gupta, R. An Optofluidic Young Interferometer for Electrokinetic Transport-Coupled Biosensing. *Micromachines 2024, Vol. 15, Page 861* **2024**, *15* (7), 861. https://doi.org/10.3390/MI15070861.

(18) Li, X.; Jia, M.; Yu, L.; Li, Y.; He, X.; Chen, L.; Zhang, Y. An Ultrasensitive Label-Free Biosensor Based on Aptamer Functionalized Two-Dimensional Photonic Crystal for Kanamycin Detection in Milk. *Food Chem* **2023**, *402*, 134239. https://doi.org/10.1016/J.FOODCHEM.2022.134239.

(19) Occhicone, A.; Sinibaldi, A.; Chiappetta, D.; Di Matteo, P.; Pileri, T.; Danz, N.; Sonntag, F.; Munzert, P.; Allegretti, M.; De Pascale, V.; Mandoj, C.; Michelotti, F. Detection of Anti-SARS CoV-2 Antibodies in Human Serum by Means of Bloch Surface Waves on 1D Photonic Crystal Biochips. *Biosens Bioelectron X* **2023**, *15*, 100413. https://doi.org/10.1016/J.BIOSX.2023.100413.

(20) Oliveira, N. C. L.; Khoury, G. El; Versnel, J. M.; Moghaddam, G. K.; Leite, L. S.; Lima-Filho, J. L.; Lowe, C. R. A Holographic Sensor Based on a Biomimetic Affinity Ligand for the Detection of Cocaine. *Sens Actuators B Chem* **2018**, *270*, 216–222. https://doi.org/10.1016/J.SNB.2018.05.009.

(21) Wang, X.; Wang, X. Aptamer-Functionalized Hydrogel Diffraction Gratings for the Human Thrombin Detection. *Chemical Communications* **2013**, *49* (53), 5957–5959. https://doi.org/10.1039/C3CC41827H.

(22) Ye, G.; Yang, C.; Wang, X. Sensing Diffraction Gratings of Antigen-Responsive Hydrogel for Human Immunoglobulin-G Detection. *Macromol Rapid Commun* **2010**, *31* (15), 1332–1336. https://doi.org/10.1002/MARC.201000082.

(23) Zhao, J. J.; Wang, W.; Wang, W.; Wang, F.; Zhao, Y.; Cai, Q. W.; Xie, R.; Xie, R.; Ju, X. J.; Ju, X. J.; Liu, Z.; Liu, Z.; Faraj, Y.; Faraj, Y.; Chu, L. Y. Smart Hydrogel Grating Immunosensors for Highly Selective and Sensitive Detection of Human-IgG. *Ind Eng Chem Res* **2020**, *59* (22), 10469–10475. https://doi.org/10.1021/ACS.IECR.0C00780/SUPPL_FILE/IE0C00780_SI_001.PDF.

(24) Juste-Dolz, A.; Delgado-Pinar, M.; Avella-Oliver, M.; Fernández, E.; Pastor, D.; Andrés, M. V.; Maquieira, Á. BIO Bragg Gratings on Microfibers for Label-Free Biosensing. *Biosens Bioelectron* **2021**, *176*, 112916. https://doi.org/10.1016/J.BIOS.2020.112916.

(25) Juste-Dolz, A.; Fernández, E.; Micó, G.; Bru, L. A.; Muñoz, P.; Avella-Oliver, M.; Pastor, D.; Maquieira, Á. Surface Bragg Gratings of Proteins Patterned on Integrated Waveguides for (Bio)Chemical Analysis. *Microchimica Acta* **2024**, *191* (1), 1–11. https://doi.org/10.1007/S00604-023-06124-Z/FIGURES/5.

(26) Lucío, M. I.; Montoto, A. H.; Fernández, E.; Alamri, S.; Kunze, T.; Bañuls, M. J.; Maquieira, Á. Label-Free Detection of C-Reactive Protein Using Bioresponsive Hydrogel-Based Surface Relief Diffraction Gratings. *Biosens Bioelectron* **2021**, *193*, 113561. https://doi.org/10.1016/J.BIOS.2021.113561.

(27) Fuchs, Y.; Soppera, O.; Mayes, A. G.; Haupt, K. Holographic Molecularly Imprinted Polymers for Label-Free Chemical Sensing. *Advanced Materials* **2013**, *25* (4), 566–570. https://doi.org/10.1002/ADMA.201203204.

(28) Calvo-Lozano, O.; Sierra, M.; Soler, M.; Estévez, M. C.; Chiscano-Camón, L.; Ruiz-Sanmartin, A.; Ruiz-Rodriguez, J. C.; Ferrer, R.; González-López, J. J.; Esperalba, J.; Fernández-Naval, C.; Bueno, L.; López-Aladid, R.; Torres, A.; Fernández-Barat, L.; Attoumani, S.; Charrel, R.; Coutard, B.; Lechuga, L. M. Label-Free Plasmonic Biosensor for Rapid, Quantitative, and Highly Sensitive COVID-19 Serology: Implementation and Clinical Validation. *Anal Chem* **2022**, *94* (2), 975–984. https://doi.org/10.1021/ACS.ANALCHEM.1C03850/ASSET/IMAGES/LARGE/AC1C03850_0007.JPEG.

(29) Adi, W.; Biswas, D.; Shelef, M. A.; Yesilkoy, F. Multiplexed COVID-19 Antibody Quantification from Human Sera Using Label-Free Nanoplasmonic Biosensors. *Biomedical Optics Express, Vol. 13, Issue 4, pp. 2130-2143* **2022**, *13* (4), 2130–2143. https://doi.org/10.1364/BOE.454919.

(30) Loyez, M.; Lobry, M.; Hassan, E. M.; DeRosa, M. C.; Caucheteur, C.; Wattiez, R. HER2 Breast Cancer Biomarker Detection Using a Sandwich Optical Fiber Assay. *Talanta* **2021**, *221*, 121452. https://doi.org/10.1016/J.TALANTA.2020.121452.

(31) Cubells-Gómez, A.; Lucío, M. I.; Bañuls, M.-J.; Maquieira, Á. Holographic Surface Relief Diffraction Gratings Made of Hydrogels for Direct Label-Free Biosensing of IgGs. *Talanta* **2024**, *279*, 126563. https://doi.org/10.1016/j.talanta.2024.126563.
